# Microbes strike back: End of golden era of antibiotics?

**DOI:** 10.4103/0970-1591.70559

**Published:** 2010

**Authors:** Nitin Kekre

**Affiliations:** Department of Urology, Christian Medical College, Vellore - 632 004, Tamil Nadu, India. E-mail: editor@indianjurol.com

Alexander Fleming was awarded the Nobel Prize for the discovery of penicillin. This discovery changed the course of modern medicine and surgery. Many infections which were untreatable became treatable. Surgical infections could be effectively treated, and surgical procedures became safer. Guidelines were developed for use of antibiotics. Most of the clean procedures can be performed without prophylaxis, as long as aseptic techniques & sterilization are strictly followed. But unfortunately many preferred indiscriminate use of antibiotics to prevent infections. Sadly today the antibiotic resistance has become a serious threat. The golden era of last century appears to be ending and the micro organisms are slowly and surely winning the battle. Last month, Indian media was busy in reporting the findings of a paper published in the Lancet Infectious Disease Journal. This was a collaborative study between UK and India on antimicrobial resistance and it reported about the origin of the new antibiotic-resistant bacteria named as New Delhi Metallo Beta Lactamace gene (NDMI). This gene is located to a large extent in *Escherichia coli (E. coli). E. coli* is the source of community acquired infections particularly of urinary tract and multi drug resistance to *E. coli* is not a new phenomenon. And according to this paper, the main reservoir of this gene is located in India, Pakistan and Bangladesh. So the threat that this drug-resistant bacterium would spread in India is very real, especially when we lack basic sanitation. Ironically, most of our hospitals and doctors do not pay attention to infection control. So it was not surprising that Indian media and a particular group of physicians were busy in portraying this as a conspiracy against India and its emergence as a destination of medical tourism.

These so called experts (who are commonly seen on English TV channels) never accepted the growing problem of antimicrobial resistance or the fact that in this country antibiotics are hugely misused. It is not uncommon to see the first urine culture report in a hospital to be resistant to most of the first line antibiotics. Methicillin-resistant *Staphylococcus aureus* (MRSA) has routinely become carbapenem-resistant and is increasing every day and Meropenem has become the antibiotic of choice. General public can get antibiotics even without a prescription and self medication with antibiotics for diarrhea and common cold has become a norm. Most of the doctors (including the physician of alternative medicine) are routinely prescribing antibiotics. We had conducted a survey among the urologists of India about percutaneous nephro lithotomy (PCNL) to audit routine clinical practice. Majority reported that they do not obtain urine culture but prefer to put them on broad spectrum antibiotic for long duration. This is due to lack of proper protocol and guidelines about the usage of antibiotics. Most of us still wrongly think that antibiotics would reduce the risk of infection. Basic sound aseptic technique, good sterilization, limited and appropriate use of antibiotics would have prevented this alarming situation. Compare this to united kingdom, where antibiotics in common use have remained the same over the years: Trimethoprim and Amoxycillin for UTI and Inj. Gentamycin for prophylaxis in urological procedure. Other broad spectrum antibiotics can only be used if indicated by culture and require microbiologists consent. It would be better if we audit our practice and evolve a national concensus and develop guidelines which would limit the use of antibiotics. Antimicrobial resistance (AMR) problem is a serious threat and it should be tackled on a war footing. The sale of antibiotics without a prescription must be stopped. All antibiotic prescriptions should be periodically audited. Alexander Fleming actually predicted the future and possibility of AMR when he said “The time may come when penicillin can be bought by anyone in the shops. Then there is a danger that the ignorant man may easily under dose himself and by exposing his microbes to non lethal quantities of the drug make them resistant”. The only difference is that in our set up the learned are more responsible for the AMR than the ignorant.

This issue has a special feature on newer instrumentation and development in Endourology. It deals with newer trends in the field of minimally invasive surgery. Dr. Manoj Monga, Director, Steven Streem Center for Endourology and Stone Disease the Cleveland Clinic Foundation Cleveland, -USA and his colleagues have done a wonderful job. I thank him and his contribution for the excellent work.

With best wishes

